# MKK3 Cascade Regulates Seed Dormancy Through a Negative Feedback Loop Modulating ABA Signal in Rice

**DOI:** 10.1186/s12284-023-00679-4

**Published:** 2024-01-03

**Authors:** Xingxue Mao, Xiaoyu Zheng, Bingrui Sun, Liqun Jiang, Jing Zhang, Shuwei Lyu, Hang Yu, Pingli Chen, Wenfeng Chen, Zhilan Fan, Chen Li, Qing Liu

**Affiliations:** 1 Rice Research Institute, Guangdong Academy of Agricultural Sciences, Guangzhou, 510640 China; 2 Key Laboratory of Genetics and Breeding of High Quality Rice in Southern China (Co-Construction By Ministry and Province), Ministry of Agriculture and Rural Affairs, Guangzhou, 510640 China; 3 Guangdong Key Laboratory of New Technology in Rice Breeding, Guangzhou, 510640 China; 4 Guangdong Rice Engineering Laboratory, Guangzhou, 510640 China

**Keywords:** Rice (*Oryza sativa* L.), Germination, MKK3 cascade, ABA

## Abstract

**Background:**

With the increasing frequency of climatic anomalies, high temperatures and long-term rain often occur during the rice-harvesting period, especially for early rice crops in tropical and subtropical regions. Seed dormancy directly affects the resistance to pre-harvest sprouting (PHS). Therefore, in order to increase rice production, it is critical to enhance seed dormancy and avoid yield losses to PHS. The elucidation and utilization of the seed dormancy regulation mechanism is of great significance to rice production. Preliminary results indicated that the OsMKKK62-OsMKK3-OsMPK7/14 module might regulate ABA sensitivity and then control seed dormancy. The detailed mechanism is still unclear.

**Results:**

The overexpression of *OsMKK3* resulted in serious PHS. The expression levels of *OsMKK3* and *OsMPK7* were upregulated by ABA and GA at germination stage. OsMKK3 and OsMPK7 are both located in the nucleus and cytoplasm. The dormancy level of double knockout mutant *mkk3/mft2* was lower than that of *mkk3*, indicating that *OsMFT2* functions in the downstream of MKK3 cascade in regulating rice seeds germination. Biochemical results showed that OsMPK7 interacted with multiple core ABA signaling components according to yeast two-hybrid screening and luciferase complementation experiments, suggesting that MKK3 cascade regulates ABA signaling by modulating the core ABA signaling components. Moreover, the ABA response and ABA responsive genes of *mpk7/14* were significantly higher than those of wild-type ZH11 when subjected to ABA treatment.

**Conclusion:**

MKK3 cascade mediates the negative feedback loop of ABA signal through the interaction between OsMPK7 and core ABA signaling components in rice.

**Supplementary Information:**

The online version contains supplementary material available at 10.1186/s12284-023-00679-4.

## Background

Seed dormancy inhibits seed germination under adverse environment conditions and increases the survival and fitness of wild plants. For rice, dormancy affects the consistency of seedling emergence and the resistance to pre-harvest sprouting (PHS). The consistency of seedling emergence affects the yield, and the resistance to PHS affects the final harvest yield and quality, both of which are important links in crop production (Shu et al. 2016). For rice production, moderate levels of seed dormancy are favorable. Dormancy is inadvertently lost in the long-term breeding process. Uncovering the mechanisms driving dormancy regulation is critical for rice production and yield optimization, particularly by enhancing moderate dormancy in seeds. ABA and GA are the primary hormones controlling seed dormancy, and the ABA/GA balance determines dormancy or germination. The ABA signal promotes the production and maintenance of seed dormancy (Shu et al. 2016; Kucera et al. 2005). ABA signaling is determined by ABA content and ABA sensitivity. ABA content is mainly controlled by NCED (9-cis-epoxycarotenoid dioxygenase), catalyzing the limiting steps of ABA synthesis, and ABA8OX (ABA8'-hydroxylase, also known as Cytochrome P450 Monooxygenase, CYP707A), which regulates the limiting steps in degradation (Lefebvre et al. 2006). ABA sensitivity is mainly related to the generation mechanism of ABA signal. *PYR/PYL/RCAR-PP2C-SnRK2*, the generation pathway of an ABA signal, is widely accepted (Fujii et al., 2009). *TaMFT* has been proved to control seed dormancy (Liu et al. 2015; Nakamura et al. 2011), which interacts with OsbZIP23/66/72 and regulates the downstream ABA responsive genes (Song et al. 2020). *OsMFT2,* the homolog of *TaMFT* in rice, was negatively regulated by MKK3 cascade (Mao et al. 2019). Further investigation is necessary in order to clarify the regulatory mechanism of the MKK3 cascade on seed dormancy.

The MAPK cascade is an evolutionarily conserved signaling module that plays diverse roles in plants (Rodriguez et al. 2010), which magnifies the signal through sequential phosphorylation of downstream proteins, integrating the information between the surrounding environment and the metabolic response centers. *OsMKK3* was induced by mechanical wounding, infestation of *Xanthomonas oryzae*, and treatments with methyl jasmonate or salicylic acid, suggesting that it could play diverse roles in different conditions (Jalmi and Sinha 2016; Zhou et al. 2019; Sözen et al. 2020). Phenotype analysis results also showed that *OsMKK3*/*OrMKK3* influenced seedling growth, grain size, and eating quality (Pan et al. 2021a, b). MKK3 is effectively a multi-signal distribution and switching center. It can mediate ABA, hydrogen peroxide and light signals, which may be involved in dormancy regulation (Dóczi et al. 2007; Sethi et al. 2014; Danquah et al. 2015). Multiple MAPK genes have been proved to regulate seed dormancy, which expanded our understanding in dormancy research. MKK1–MAPK6 positively affects seed dormancy and the expression of *AtCAT1* in *Arabidopsis* (Xing et al. 2009, 2008). In *Arabidopsis,* two MKKK genes, *AtRAF10* and *AtRAF11*, controlled seed dormancy by regulating the expression of *AtABI3* and *AtABI5 *(Lee et al. 2015), which were key genes in ABA response (Du et al. 2018; Hobo et al. 1999; Zou et al. 2008).

Overexpression of *OsMKKK62* resulted in the loss of dormancy in rice. Knockouts of the downstream members, *OsMKK3*, *OsMPK7,* and *OsMPK14*, lead to the evident increase of seed dormancy level, indicating that MKK3 cascade might substantially influence ABA signaling (Mao et al. 2019). According to sequence similarity, *AtMKKK14/15/16/17/18-AtMKK3-AtMPK1/2/7/14* should function like *OsMKKK62-OsMKK3-OsMPK7/14* (MKK3 cascade) (Rao et al. 2010; Hamel et al. 2006). ABA induced the expression of *AtMKKK18* and promoted stomatal closure (Mitula et al. 2015). PP2C protein, ABI1, interacted with At MKKK18 and promoted its degradation (Mitula et al. 2015). One group also reported the interaction between ABI1 and *AtMKKK18* (Choi et al. 2017). Another finding demonstrated the activation of AtMPK7 induced by ABA was inhibited in the mutants, *atmkk3-1* and *atmkkk17/18* (Danquah et al. 2015). These results indicated that the MKK3 cascade might take part in the ABA signal. The homolog gene of *AtMKK3* controlled seed dormancy in barley and wheat (Nakamura et al. 2016; Torada et al. 2016). In rice, the MKK3 cascade negatively regulated ABA sensitivity and seed dormancy (Mao et al. 2019). In *Arabidopsis*, similar phenomena have been observed (Danquah et al. 2015; Mitula et al. 2015; Choi et al. 2017), although the regulatory effect in *Arabidopsis thaliana* was smaller than that in rice, suggesting that the MKK3 cascade influences ABA signaling greatly.

In this study, we explored the expression characteristics of MKK3 cascade members, checked the germination phenotype of related mutants, identified several OsMPK7 interaction proteins, and finally proposed that MKK3 cascade mediated the negative feedback loop of ABA signaling in rice.

## Results

### Overexpression of* OsMKK3* Resulted in PHS and Decreased the Tolerance to Dehydration

**Fig. 1:**
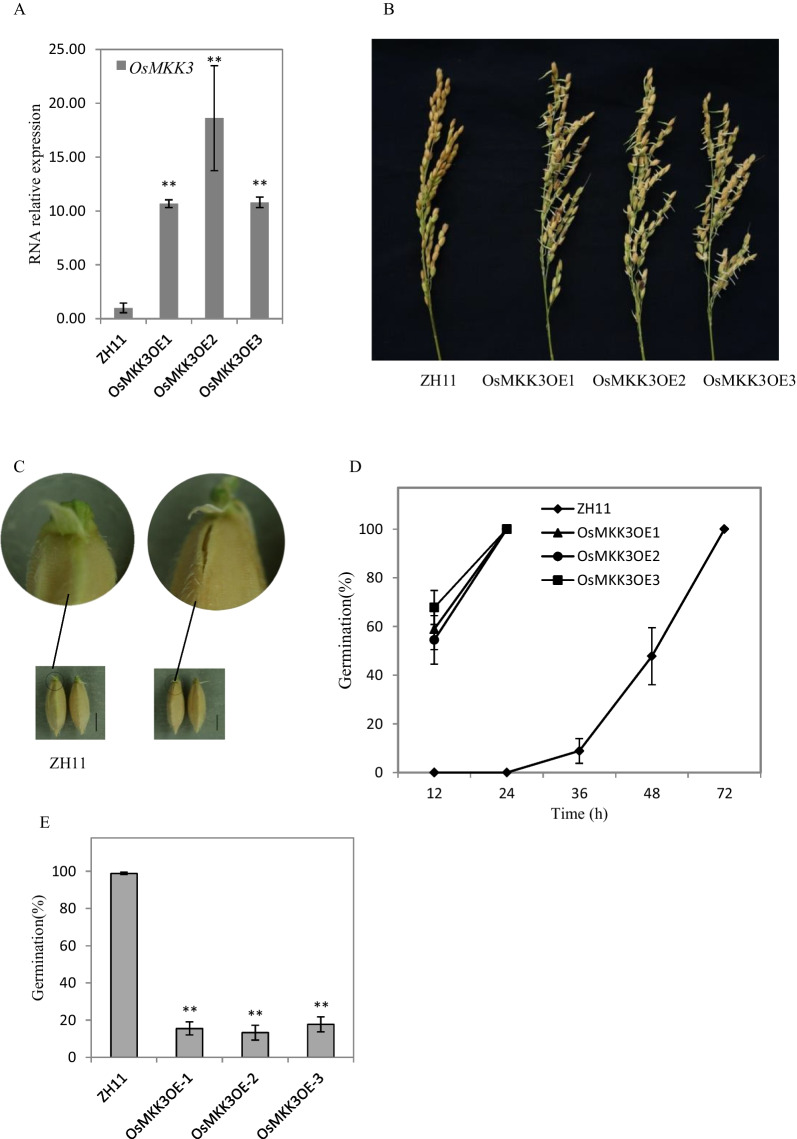
Overexpression of *OsMKK3* leads to PHS and the loss of dehydration tolerance. **A** Relative expression level of *OsMKK3* in leaves of OsMKK3OE lines. Values represent the mean ± SD of three biological replicates. Student’s t-tests were used to generate *p *values. (** = *p* < 0.01) **B** Germination phenotype of the OsMKK3OE panicles. After 25 days of heading, panicles of overexpression lines and ZH11 were sampled for germination tests, and the photos were taken after two days of treatment. **C** After 25 days of heading, small cracks appeared in the embryonic part of OsMKK3OE husk (Scale bar = 2 mm). **D** Freshly harvested seeds were used for germination test, and germination percentage was calculated at the indicated time. Values represent the mean ± SD of three biological replicates (30 seeds for each replicate). **E** The germination rates of the dry seeds were calculated after four days germination treatment. Values represent the mean ± SD of three biological replicates (30 seeds for each replicate). Student’s t-tests were used to generate *p *values. (** = *p* < 0.01)

The overexpression of *AtMKK3* did not lead to vivipary in *Arabidopsis thaliana* (Danquah et al. 2015). To further check the function of *OsMKK3*, we created Os*MKK3*-overexpressing (OsMKK3OE) transgenic lines. *OsMKK3* was under the control of the maize ubiquitin promoter (*Ubi*:*OsMKK3*). By checking the hygromycin resistance at germination stage, three homozygous lines (OsMKK3OE1, OsMKK3OE2, OsMKK3OE3) were selected for characterization. The gene expression analysis results showed that the transcript levels of *OsMKK3* were higher in leaves of the three OsMKK3OE lines than that in ZH11 at three-leaf stage (Fig. 1A). To test the germination character, we harvested the panicles and treated them under germination conditions for two days. All seeds from overexpression lines germinated, but no wild-type ZH11 seeds germinated (Fig. 1B). At 25 days after heading, small cracks appeared in the embryonic part of OsMKK3OE husk (Fig. 1C), indicating that the germination process began before full maturity even without rain. A germination experiment showed that OsMKK3OE seeds could germinate more quickly than wild-type ZH11 seeds (Fig. 1D). To evaluate the influence of dehydration tolerance, we carried out the germination experiment after drying (50 °C, three days). The germination rate of OsMKK3OE seeds decreased substantially, while the wild-type ZH11 germinated normally (Fig. 1E), suggesting that dehydration tolerance decreased greatly in OsMKK3OE seeds.

### Organizational Expression and Subcellular Localization and of OsMKKK62, OsMKK3 and OsMPK7

**Fig. 2:**
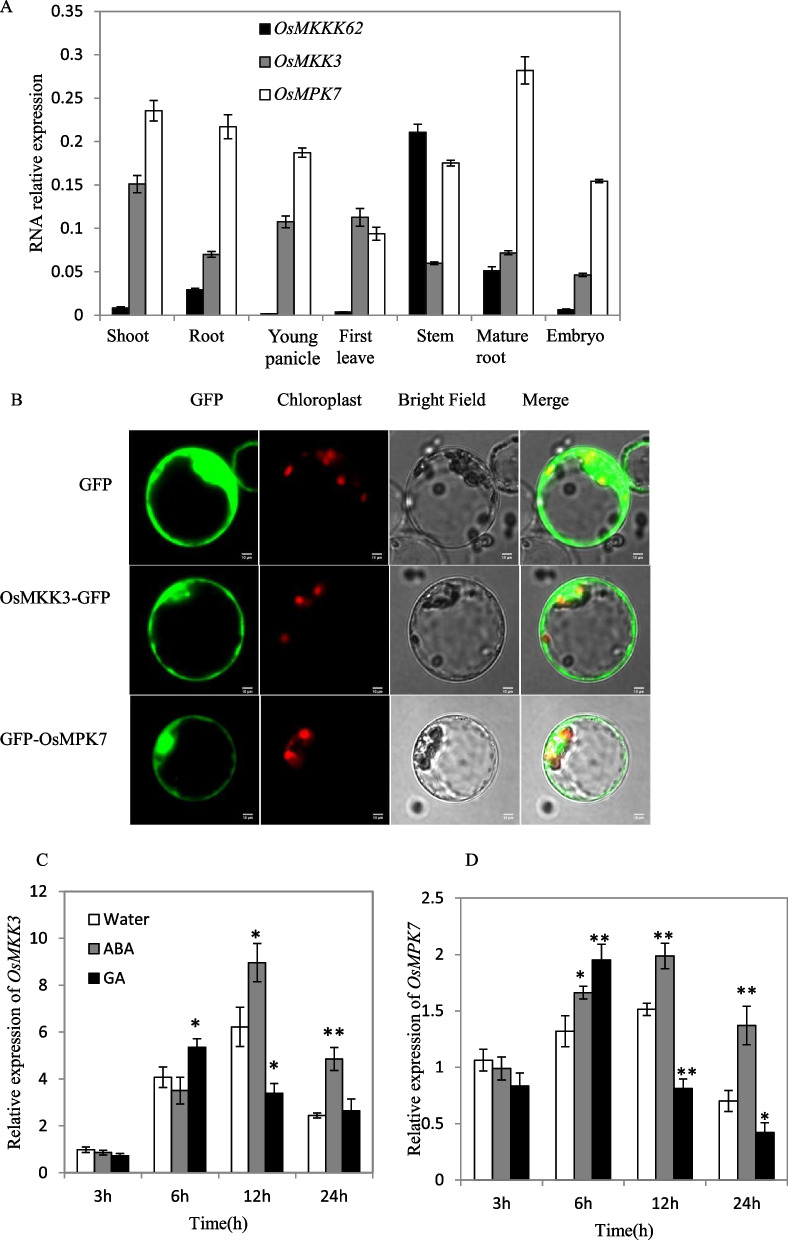
Expression characteristics of *OsMKKK62*, *OsMKK3* and *OsMPK7.* **A** Transcription analysis of *OsMKKK62*, *OsMKK3* and *OsMPK7* in different tissues by quantitative RT-PCR. Shoot and root were sampled at three-leaf stage; young panicle, first leaf, stem and mature root were sampled before heading; embryos were sampled at the late period of dough stage. Values represent the means ± SD of three biological replicates. **B** Subcellular localization of OsMKK3 and OsMPK7 in rice protoplasts. GFP, OsMKK3-GFP and GFP-OsMPK7 driven by the 35S promoter under green fluorescence, red fluorescence, bright field, and merged views (Scale bar = 10 μm). **C**, **D** The expression of *OsMKK3* (**C**) and *OsMPK7* (**D**) in embryos treated with ABA or GA. The seeds were treated with 100 μM ABA, 100 μM GA and water respectively. After 3, 6, 12, and 24 h of treatment, ten embryos were cut for gene expression analysis. Values represent the mean ± SD of three biological replicates. Student’s t-tests were used to generate *p *values. (* = *p* < 0.05, ** = *p* < 0.01)

To investigate the biological role of *OsMKK3* in rice, we analyzed the spatial and temporal expression of *OsMKK3* in different rice tissues. *OsMKK3* was ubiquitously expressed in the whole plant, and the transcription levels were relatively high in the shoot of the three-leaf stage, the first leaf and the young panicle before heading (Fig. 2A). We also analyzed the expression of *OsMPK7* and *OsMKKK62* with a similar method, showing that *OsMPK7* was also widely expressed in the whole plant, the transcription levels were relatively high in young panicles and shoots and *OsMKKK62* was mainly expressed in root and stem (Fig. 2A). The subcellular localization of OsMKK3 was determined through transient expression of an OsMKK3-GFP fusion protein in rice protoplasts. Fluorescence from the fusion protein was observed in the nuclei and cytoplasm (Fig. 2B), indicating that OsMKK3-GFP was located in both. To further learn the function of the MKK3 cascade, we checked the subcellular localization of OsMPK7 and OsMKKK62. With the same method, OsMPK7-GFP and OsMKKK62-GFP did not show a clear signal. To further check the subcellular localization of OsMPK7, we constructed another fusion vector, GFP-OsMPK7, introduced it in the protoplasts, and observed the bright fluorescence signal at nuclei and cytoplasm (Fig. 2B). These results indicated that MKK3 cascades may function in both nuclei and cytoplasm.

### ABA and GA Induced the Expression of* OsMKK3* and *OsMPK7*

ABA and GA are the primary hormones controlling seed dormancy. To identify the induction effect of ABA and GA on MKK3 cascades, we treated the seeds with ABA and GA solution, and performed expression analysis with the embryos. The results showed that both ABA and GA could induce the expression of *OsMKK3* and *OsMPK7*, the induction effect of ABA lasted for a long time, and the induction effect of GA disappeared quickly (Fig. 2C, D). The induction effect of ABA or GA on *OsMKKK62* is not obvious (Data not shown). These results indicated that MKK3 cascade may be involved in the signal transmission of both ABA and GA.

### Hydrogen Peroxide Could Promote the Germination of *mpk7/14*

**Fig. 3:**
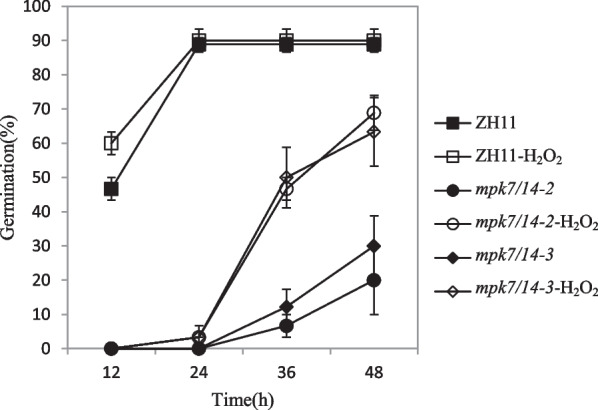
Germination result of *mpk7/14*in hydrogen peroxide. Before germination test, all seeds were dehusked. The seeds were treated with 20 mM hydrogen peroxide and water as control. At the indicated time the germinated seeds were counted. Values represent the mean ± SD of three biological replicates (30 seeds for each replicate)

Biochemical experimental results showed that the MKK3 cascade may be involved in the signal of hydrogen peroxide in *Arabidopsis* (Dóczi et al. 2007). Hydrogen peroxide is a molecular hub of ROS signal and often acts as a rice germination promoter (Chen et al. 2016; Peng et al. 2022). We tested the germination of *mpk7/14*with exogenous 20 mM hydrogen peroxide. In order to eliminate the effect of residual hydrogen peroxide on germination, all seeds were dehusked. The results indicated that hydrogen peroxide could obviously promote the germination of *mpk7/14* (Fig. 3).

### Knockout of *OsMFT2* Could Partially Rescue the Germination Phenotype of *mkk3* Mutant

**Fig. 4:**
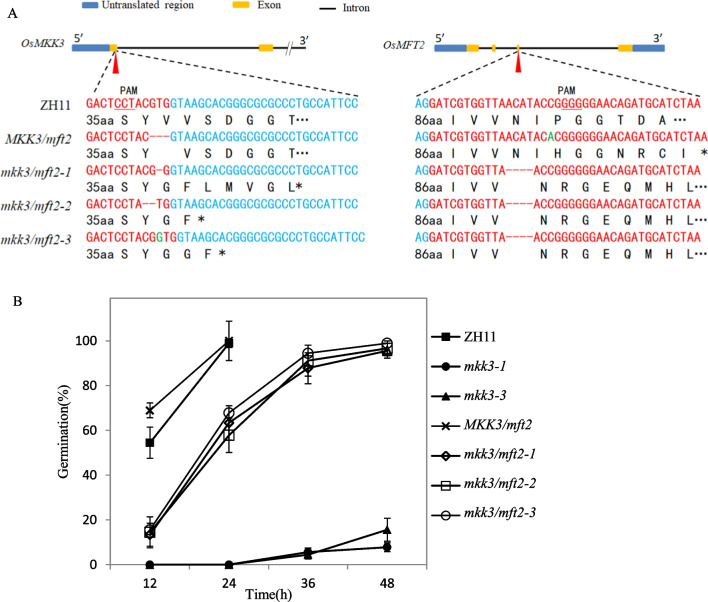
Germination phenotype of Crispr lines. **A** Schematic of four Crispr lines. Red letters indicate the exon sequences, blue letters indicate the intron sequences, green letters indicate the inserted nucleotide and black letters indicate amino acids. Dashs indicate the deleted nucleotides. Asterisks indicate termination codons. PAM, protospacer adjacent motif. Red triangles indicate the target position. **B** Germination phenotype of *OsMKK3/OsMFT2* Crispr lines. The germination rates were calculated at 12 h intervals. Values represent the mean ± SD of three biological replicates (30 dehusked seeds for each replicate)

Expression analysis showed that *OsMFT2* was negatively regulated by MKK3 cascade (Mao et al. 2019). *AtMFT*, the homologous gene of *OsMFT2 in Arabidopsis,* was a vital gene in the ABA signal pathway (Xi et al. 2010). To check the functional relationship between the MKK3 cascade and *OsMFT2*, we constructed CRISPR vector to edit *OsMFT2* and *OsMKK3* simultaneously, and introduced the vector into ZH11 through *Agrobacterium*-mediated genetic transformation. According to the sequencing chromatogram of the T_2_ generation (Additional file 1: Fig. S1), four independent Crispr lines were screened for planting (Fig. 4A) and the transgenic seeds were dehusked for a germination test. *MKK3/mft2* (3 nucleotide deletion in *OsMKK3,* 1 nucleotide insertion in *OsMFT2*) showed a germination phenotype similar to that of wild-type ZH11. The mutation of *OsMKK3* inhibited seed germination (Mao, et al. 2019). *OsMFT2* negatively regulated seed germination (Song et al. 2020). The germination performance of *MKK3/mft2* indicated that the 3-nucleotide deletion in *OsMKK3* did not affect its function. The germination performance of *MKK3/mft2* could represent the phenotype of the *osmft2* mutant. When *OsMKK3* and *OsMFT2* were knocked out simultaneously (*mkk3/mft2-1, mkk3/mft2-2, mkk3/mft2-3*), the germination rates were significantly higher than that of *mkk3* (Fig. 4B). The result suggested that the mutation of *OsMFT2* could partially rescue the germination phenotype of *osmkk3* mutant, and MKK3 cascade possibility acted on the upstream of *OsMFT2* in the dormancy regulation pathway.

### Identification of Interaction Proteins of OsMPK7

**Fig. 5:**
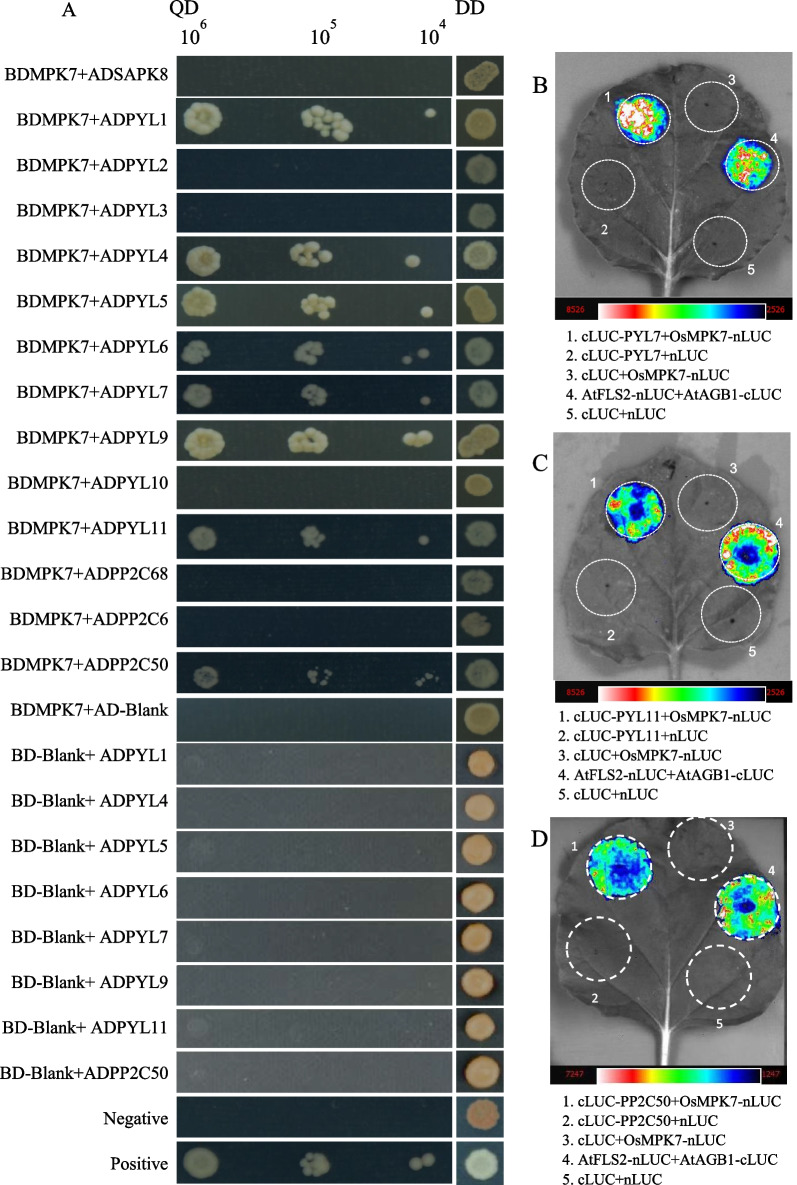
Interaction analysis between OsMPK7 and core ABA signaling components. **A** Y2H assay of interactions between OsMPK7 and ABA signaling precursors. QD (Quadruple-dropout medium lacking Ade, His, Leu and Trp); DD, (Double-dropout medium lacking Leu and Trp). The numbers above represent the cell concentration (cells/mL), and the proteins expressed in the yeast are listed on the left. **B**–**D** Luciferase complementation results of OsMPK7’s interaction with OsPLY7, OsPLY11 and OsPP2C50 in *N. benthamiana* leaves

ABA is the primary hormone that regulates seed dormancy and germination, which regulates ABA responsive genes through core ABA signaling components (PYR/PYL/RCAR-PP2C-SnRKs) (Cutler et al. 2010; Hubbard et al. 2010). In *Arabidopsis*, *Snrk2.2/2.3/2.6* play vital roles in ABA signal transduction and the triple mutant exhibited a loss of seed dormancy (Nakashima et al. 2009; Fujii and Zhu 2009). As the homologs of *Snrk2.2/2.3/2.6* in rice, OsSAPK8/9/10 were also activated by ABA, suggesting their functions in dormancy regulation (Kobayashi et al. 2004). Among these three genes, the expression level of *OsSAPK8* is the highest in later stages of maturation (Sato et al. 2013), suggesting its crucial function in seed dormancy. We were interested in whether OsMPK7 could interact with OsSAPK8 (Fig. 5A). To test this, we performed the Y2H experiment. According to the growth on QD, OsMPK7 did not interact with OsSAPK8. With the same method, we checked interaction relationships of OsMPK7 with 10 OsPLYs and 3 OsPP2Cs. The results showed that OsMPK7 could interact with 6 OsPLYs and OsPP2C50 (Fig. 5A). To further confirm these interactions, we selected OsPLY7, OsPLY11 and OsPP2C50 to perform Luciferase Complementation experiments. Chemiluminescence imaging results also supported the interaction of OsMPK7 with these three proteins (Fig. 5B–D). All the interaction results indicated that the MKK3 cascade regulated ABA signal through the interaction between OsMPK7 and core ABA signaling components.

### ABA-Response was Upregulated in *mpk7/14*

**Fig. 6:**
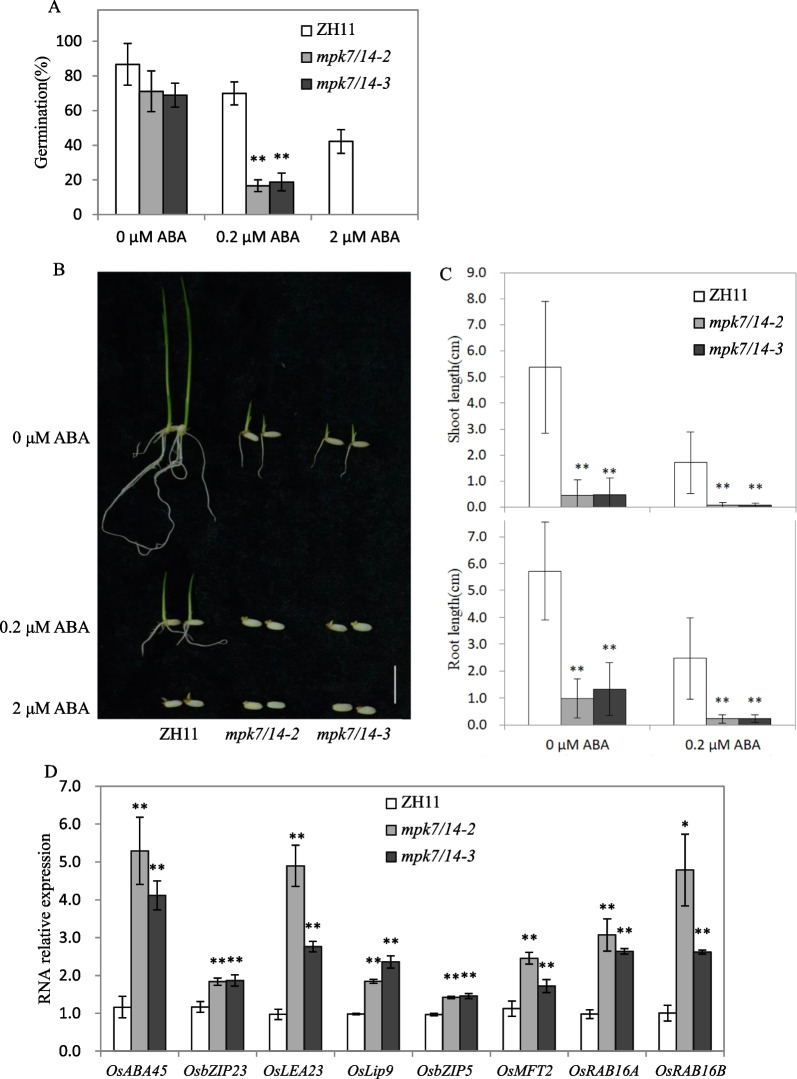
Mutant *mpk7/14* showed enhanced ABA response. **A** The germination performance of ZH11 and *mpk7/14*. The germinated seeds were counted after 6 days of ABA treatment, Values represent the mean ± SD of three biological replicates (30 seeds for each replicate). The asterisks indicate significant differences compared with ZH11. Student’s t-tests were used to generate *p* values (*p < 0.05, **p < 0.01). **B** Phenotypes of ZH11and *mpk7/14* after 6 days of ABA treatment on 1/2 MS culture medium without or with ABA (0.2 μM or 2 μM) (Scale bar = 1 cm). **C** The lengths of shoots and roots of ZH11 and *mpk7/14* after 6 days of ABA treatment. Values represent the mean ± SD of three biological replicates (10 germinated seeds for each replicate). The asterisks indicate significant differences compared with mock treatment. Student’s t-tests were used to generate *p* values (**p < 0.01). **D** Expression analysis of ABA responsive genes in ZH11 and *mpk7/14*. Seeds were soaked in water for six hours at 30 °C and ten embryos were cut for gene expression analysis. Values represent the mean ± SD of three biological replicates. The asterisks indicate significant differences compared with ZH11. Student’s t-tests were used to generate *p* values (*p < 0.05, **p < 0.01)

A batch of interactions between OsMPK7 and the core ABA signaling component indicated the close relationship between the MKK3 cascade and the ABA signal. To evaluate the effect of ABA on *mpk7/14* mutant, *mpk7/14* seeds were treated with ABA for six days. Under mock treatment (0 μM ABA), there was no significant difference in germination rate between ZH11 and *mpk7/14*, but under 0.2 μM ABA, the germination rates of *mpk7/14* seeds (15.6% and 17.8%) were extremely significantly lower than that of ZH11 (70%) (Fig. 6A). Under 2 μM ABA, the germination rate of ZH11 was 42.2%, and all *mpk7/14* seeds could not germinate (Fig. 6A, B). Moreover, the shoot lengths and root lengths of ZH11 were significantly longer than those of *mpk7/14* without or with ABA treatment (Fig. 6C). The root lengths of ZH11, *mpk7/14–2* and *mpk7/14–3* under 0.2 μM ABA treatment were 43.0%, 23.8% and 17.2% of those under mock treatment, respectively (Additional file 2: Fig. S2). The inhibition effect of 0.2 μM ABA on *mpk7/14* was significantly stronger than that on ZH11, suggesting that *mpk7/14* was more sensitive to ABA than that of ZH11.

To further check this deduction, we selected several ABA responsive genes (Tian et al. 2015; Zang et al. 2016; Mukherjee et al. 2006; Wang et al. 2022) and compared their expression levels in *mpk7/14* and wild-type ZH11. The results showed that the expression levels of ABA responsive genes in *mpk7/14* were significantly higher than those in ZH11 (Fig. 6D), suggesting that MKK3 cascade negatively regulates ABA signal.

## Discussion

The consistency of germination affects seedling raising, and the resistance to PHS affects the quality of harvested seeds, both of which are important for crop production. Seed dormancy negatively regulates consistent germination and positively regulates PHS tolerance. Moderate seed dormancy is suitable for crop production.

*OsMKKK62* and *LOC103652526* (the maize homolog of *OsMKKK62*) positively regulate the germination of rice (Mao et al. 2019), indicating MKK3 cascade plays an important role in seed germination in *Gramineae*. The overexpressed *OsMKK3* resulted in PHS and further confirmed that the MKK3 cascade negatively regulated seed dormancy (Fig. 1B). Overexpression of *AtMKK3* showed little effect on germination in *Arabidopsis* (Danquah et al. 2015). This suggests stark differences in the dormancy regulation mechanism between rice and *Arabidopsis*.

**Fig. 7:**
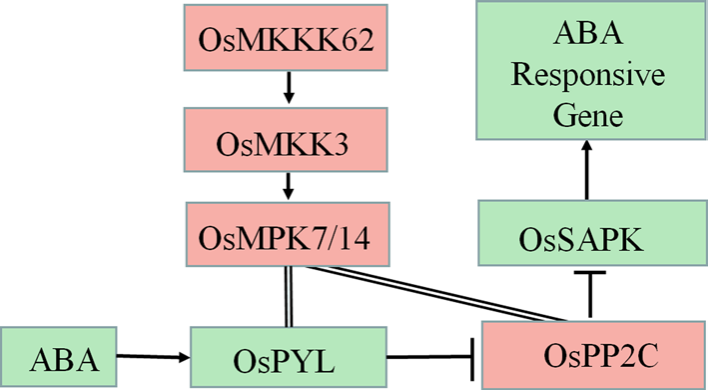
Working model of MKK3 cascade integrating core ABA signaling. Green frames indicate positive regulatory factors of ABA signals, and red frames indicate negative regulatory factors. Arrows indicate positive regulation; flat-ended arrows indicate negative regulation; double lines indicate protein interactions

The results from *Arabidopsis* showed that the *AtMKKK17/18*, the homologous gene of *OsMKKK62*, was induced by ABA in leaves (Matsuoka et al. 2015; Mitula et al. 2015; Danquah et al. 2015). Our results showed that ABA and GA could induce the expression of *OsMKK3* and *OsMPK7* in germinating seeds (Fig. 2C, D). ABA and GA antagonistically regulate seed germination (Shu et al. 2016). MKK3 cascade positively regulates germination (Fig. 1B) (Mao et al. 2019). There should be an interesting mechanism governing this contradictory phenomenon among ABA, GA, and MKK3 cascades. The expression of *AtMFT* is regulated by ABA (Xi et al. 2010). *OsMFT2* is the rice homologous gene of *AtMFT* and the transcription of *OsMFT2* is regulated by MKK3 cascade (Mao et al. 2019). Knockout experiments showed that the germination rate of double mutant (*mkk3/mft2-1, mkk3/mft2-2* and *mkk3/mft2-3*) was faster than that of *mkk3* (Fig. 4B). These results indicated that MKK3 cascade may function upstream of *OsMFT2*. Both ABA and GA could regulate the expression of *AtMFT* (Xi et al. 2010). *SLR1* encodes a DELLA protein, which is a key negative regulator in GA signaling. *SLR1* mutant didn’t show serious PHS phenotype (Ikeda et al. 2001), indicating that the effect of GA on germination was not as strong as MKK3 cascade. *Snrk2.2/2.3/2.6* positively regulated ABA signal and the triple mutant showed vivipary phenotype (Nakashima et al. 2009; Fujii and Zhu 2009), which is similar with the PHS phenotype of OsMKK3OE (Fig. 1A). It indicated that MKK3 cascade may regulate seed dormancy mainly through ABA signal. ABA receptors interact with PP2C, releasing SAPK from PP2C repression and producing the ABA signal, which results in the regulation of abiotic stress resistance and growth development including germination (Park et al. 2009; Cutler et al. 2010; Klingler et al. 2010). There are 13 PLYs in rice (He et al. 2014; Yadav et al. 2020; Miao et al. 2018). We cloned 10 OsPLYs in a prey vector. Through Y2H experiments, we proved that that OsMPK7 interacted with 7 OsPLYs. In addition, we checked the interaction relationship of OsMPK7 with 3 OsPP2Cs by Y2H. The results showed that OsMPK7 interacted with OsPP2C50 (Fig. 5A). The LUC experiment also supported the interactions of OsMPK7 with OsPYL6, OsPYL11 and *OsPP2C50 *(Fig. 5B–D). Results of ABA treatment and expression analysis of ABA responsive genes also suggested that MKK3 cascade negatively regulated ABA signals (Figs. 6, Additional file 2: Fig. S2). We speculated that OsMPK7 could accept the recruitment of OsPYLs and OsPP2C, and then restrain the operation of ABA core components from producing ABA signals, thus promoting germination (Fig. 7). Recently, AtMKK3-AtMPK7-AtERF4-AtEXPA module was confirmed to regulate seed germination in *Arabidopsis thaliana* (Chen et al. 2023). Further experiments are needed to determine whether there is a similar regulatory pathway in rice.

Some PLYs existed in monomer state, produced ABA signals in the absence of ABA and ensured a basal level of ABA signaling in plant growth, while other PLYs existed in dimer state and produce ABA signal by combining molecular ABA (Hao et al. 2011; Tian et al. 2015; Dupeux et al. 2011). OsMPK7 could interact with dimer OsPYL1 or monomer OsPYL*6*, suggesting MKK3 cascade could regulate basal ABA signaling and the ABA signals induced by molecular ABA. *OsMPK7* and *OsMKK3* were induced by ABA and inhibited the ABA signals, suggesting that MKK3 cascade was in the negative feedback loop of ABA signaling (Fig. 7). In addition, GA and hydrogen peroxide could induce the expression of *OsMKK3* and *OsMPK7 *(Fig. 2C, D) (Jalmi and Sinha 2016), and hydrogen peroxide could partially rescued the germination phenotype of *mpk7/14*, so the MKK3 cascade may mediate the crosstalk among ABA, GA and hydrogen peroxide signals.

OsPP2C68 was specifically localized in the nucleus of rice protoplasts, but OsPP2C50 and OsPP2C6 were not (Min et al. 2019). The subcellular localization corresponded to the clades observed in phylogenetic trees (Min et al. 2019; Kim et al. 2012). OsPYL1-10 were localized in the nucleus and cytosol of *Nicotiana benthamiana* leaves (Tian et al. 2015). OsMPK7 was localized in the nucleus and cytosol (Fig. 2B), and interacted with OsPLY1, OsPLY4, OsPLY5, OsPLY6, OsPLY7, OsPLY9, OsPLY11 and OsPP2C50 (Fig. 6). These results suggest that the interaction between OsMPK7 and core ABA signaling components cannot be inferred from the results of subcellular localization or simple evolutionary similarity, and that MKK3 cascades may be regulating ABA signal through fine regulation of parts of core ABA signaling components, which may be formed by later evolution.

Hydrogen peroxide is a popular germination promoter (Chen et al. 2016; Peng et al. 2022). In *Arabidopsis,* hydrogen peroxide could activate AtMPK7 in an AtMKK3-mediated manner (Dóczi et al. 2007). In rice, hydrogen peroxide could upregulate the expression of *OsMPK7* (Jalmi and Sinha 2016). The positive regulation effect of MKK3 cascade on rice germination process was obvious (Fig. 1B) (Mao et al. 2019). Therefore, hydrogen peroxide was likely to play a regulatory role through MKK3 cascade. When treated with exogenous hydrogen peroxide treatment, the germination of *mpk7/14* was partially rescued (Fig. 3), suggesting that MKK3 cascade may not be the only pathway of hydrogen peroxide signal in germination. ABA could promote the production of hydrogen peroxide in guard cells (Pei et al. 2000; Wang and Song 2008). In the process of germination, the relationship between ABA and hydrogen peroxide needs further research.

## Conclusion

Our results demonstrated that MKK3 cascade mediated the negative feedback regulation of ABA signal. In the plant lifecycle, stresses and favorable conditions often occur randomly. ABA signals promote adaption to stress condition, and the MKK3 cascade may be always ready to inhibit the ABA signal and start normal growth. Both of these are beneficial for plants to quickly adapt to stressful environment or develop in favorable conditions. Based on the strong effects on ABA signaling in growth and development, MKK3 cascade may be a major participant in the negative feedback loop of ABA signaling and more related studies should be performed to reveal its working mechanism.

## Methods

### Plant Materials and Growth Conditions

All rice (*Oryza sativa*) plants used in this study were the rice cultivar ZH11 or the transgenic progeny of ZH11. The *OsMKK3* knockout line (*mkk3-1* and *mkk3-3*) and the *OsMPK7/14* knockout line (*mpk7/14-2* and *mpk7/14-3*) were created by gene edition (Mao et al. 2019).

### Development of Overexpression Plants and Crispr Plants

For construction of the *OsMKK3-*overexpression vector, the *OsMKK3* coding sequence was amplified with cDNA from ZH11 as template and inserted into a pOX vector at the *Hind*III site by recombinant cloning (the inserted gene was controlled by ubiquitin promoter). The primers used in this paper are listed in Additional file 3: Table S1. For construction of the CRISPR vector, the target sites were designed on the web (http://cbi.hzau.edu.cn/crispr/). Two target sites were selected, which were allocated in the first exon of *OsMKK3* and the third exon of *OsMFT2,* respectively. The CRISPR vector was created with guidance from the literature (Ma et al. 2015). The overexpression vector and the CRISPR vector were introduced into ZH11 by *Agrobacterium tumefaciens* (EHA105)-mediated transformation. The subsequent selection of overexpression plants and Crispr plants were performed as described in previous study (Mao et al. 2019).

### Yeast Two-Hybrid Assay

*OsMPK7* CDS was inserted into pGBKT7 vector as the bait. The cDNA obtained from ZH11 was used as the template to amplify *OsSAPK8, OsPLYs* and *OsPP2Cs* with specific primers. All the PCR products were inserted into the pGADT7 as preys, respectively. Yeast Two-Hybrid experiments were conducted with OsMPK7-bait and performed as described in previous study (Mao et al. 2019).

### Luciferase Complementation Assay

The fragment of *OsMPK7* CDS was inserted into pCAMBIA1300-nLUC vector, and the *OsPLY*s and *OsPP2C50* CDS were inserted into the pCAMBIA1300-cLUC vector, and then introduced into *Agrobacterium tumefaciens* GV3101. *Agrobacterium* containing *pCAMBIA1300-nLUC-OsMPK7* was co-injected with each *Agrobacterium* containing cLUC fusion vector into the same part of the *N. benthamiana* leaf and incubated for 48 h. After D-luciferin treatment, LUC activity was photographed using chemiluminescence imaging (FUSION FX EDGE SPECTRA).

### Germination Test

The newly harvested panicles were immediately soaked in water for 5 min, drained of water briefly, and kept in 30 °C and humidity > 95%. The germination phenotype was checked two days later.

Thirty seeds were distributed on a filter paper in a 9-cm dish, soaked with 10 mL water, and kept in an incubator (30 °C, humidity > 95%). The presence of 1 mm protrusions in the embryo was used as the standard of germination, and germinated seeds were counted every 12 h.

### Subcellular Localization

For protein subcellular localization analysis, the coding sequences of *OsMKK3* and *OsMPK7* were amplified using primers listed in Additional file 3: Table S1 and inserted into the GFP vector to produce OsMKK3*-*GFP and GFP-OsMPK7 fusion constructs. Subsequently, the fusion constructs were transformed into rice stem protoplasts as described previously (Zhang et al. 2011). After incubation in the dark for 24 h at room temperature, GFP fluorescence was detected by laser confocal microscopy (Zeiss LSM710, Germany).

### Gene Expression Analysis

In the seed treatment, high-quality ZH11 seeds were placed in a 9 cm petri dish containing 10 ml working solution. Working solution was either 100 μm ABA or 100 μM GA, with water as control. The dishes were kept in a 30 °C incubator. Ten embryos were cut at 3, 6, 12, and 24 h of treatment and stored in liquid nitrogen until RNA extraction. Total RNA was extracted from plant tissue with Trizol reagent (Invitrogen) according to the manufacturer’s instructions. Quantitative RT-PCR analysis was performed as described in a previous report (Mao et al. 2019).

### ABA Treatment

Thirty seeds were dehusked and sterilized with 75% ethanol for 30 s and 2.5% sodium hypochlorite solution for 25 min, washed three times with water and then placed on 1/2 MS culture medium without or with ABA (0.2 μM or 2 μM) for 6 days in a 27 °C constant temperature incubator.

### Accession Numbers

Sequence data from this article can be found in the Rice Genome Annotation Project or NCBI Database under the following accession numbers:

*OsMKKK62* (LOC_Os01g50420); *OsMKK3* (LOC_Os06g27890); *OsMPK7* (LOC_Os06g48590); *OsMFT2*(LOC_Os01g02120); *OsSAPK8*(LOC_Os03g55600); *OsABA45*(LOC_Os12g29400); *OsLEA3*(LOC_Os05g46480); *OsLip9*(LOC_Os02g44870); OsbZIP5 (LOC_Os01g46970); *OsbZIP23*(LOC_Os02g52780); *OsRAB16A*(LOC_Os11g26790); *OsRAB16B*(LOC_Os11g26780); *OsPYL1*(LOC_Os01g61210); *OsPYL2*(LOC_Os02g13330); *OsPYL3*(LOC_Os02g15640); *OsPYL4*(LOC_Os03g18600); *OsPYL5*(LOC_Os05g12260); *OsPYL6*(LOC_Os05g39580); *OsPYL7*(LOC_Os06g33640); *OsPYL9*(LOC_Os06g36670); *OsPYL10*(LOC_Os10g42280); *OsPYL11(*Os6g0526400); *OsPP2C6*(LOC_Os01g40094); *OsPP2C50*(LOC_Os05g46040); *OsPP2C68*(LOC_Os09G15670).

### Supplementary Information

**Additional file 1: Fig. S1.** The alignment result of target region in *OsMKK3/OsMFT2* Crispr lines.

**Additional file 2: Fig. S2.** Inhibitory effect of 0.2 μM ABA on *mpk7/14*. ABA sensitivity index (tissue length under 0.2 μM ABA treatment/length under 0 μM ABA treatment) of ZH11 and *mpk7/14* for shoots and roots. Values represent the mean ± SD of three biological replicates (10 germinated seeds for each replicate). The asterisks indicate significant differences compared with ZH11. Student’s t-tests were used to generate *p* values (*p < 0.05, **p < 0.01).

**Additional file 3: Table S1.** Primers used in this study.

## Data Availability

The datasets supporting the conclusions of this article are provided within the article and its additional files.

## Abbreviations

PHS: Pre-harvest sprouting
ABA: Abscisic acid
GA: Gibberellin
Y2H: Yeast two-hybrid
QD: Quadruple-dropout medium lacking Ade, His, Leu and Trp
MKK3 cascade: OsMKKK62-OsMKK3-OsMPK7/14
