# Supplementary figures and images for: MKK3 Cascade Regulates Seed Dormancy Through a Negative Feedback Loop Modulating ABA Signal in Rice
Source: Rice (N Y). 2024 Jan 3;17:2. doi: 10.1186/s12284-023-00679-4 (PMC10764673; doi:10.1186/s12284-023-00679-4)

*OsMKK3*

*OsMFT2*

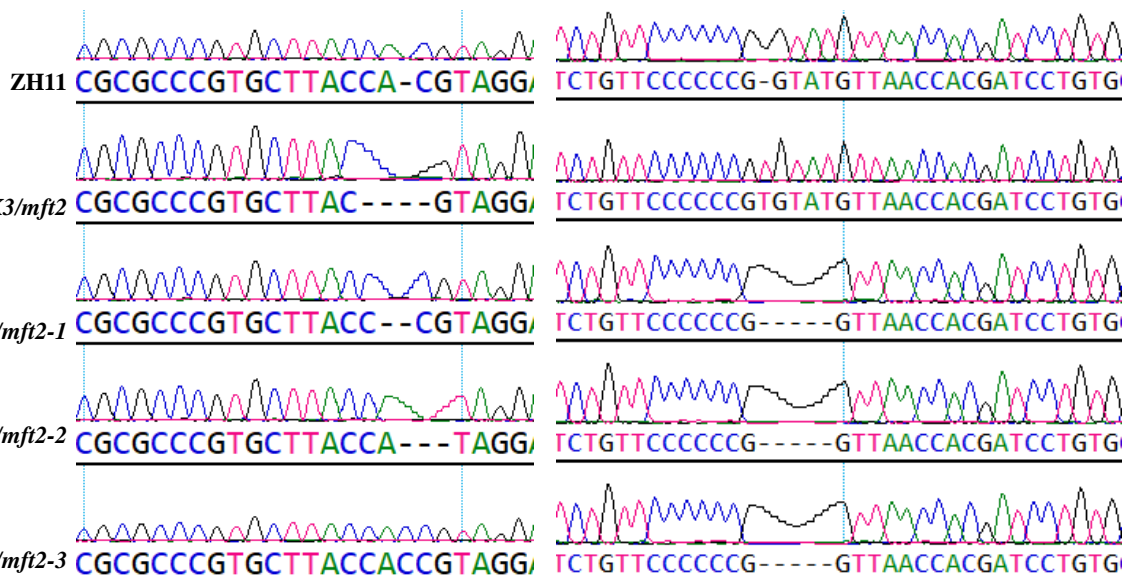

Supplement: Supplementary file 1 — Additional file 1: Fig. S1. The alignment result of target region in OsMKK3/OsMFT2 Crispr lines. [file 12284_2023_679_MOESM1_ESM.pdf]

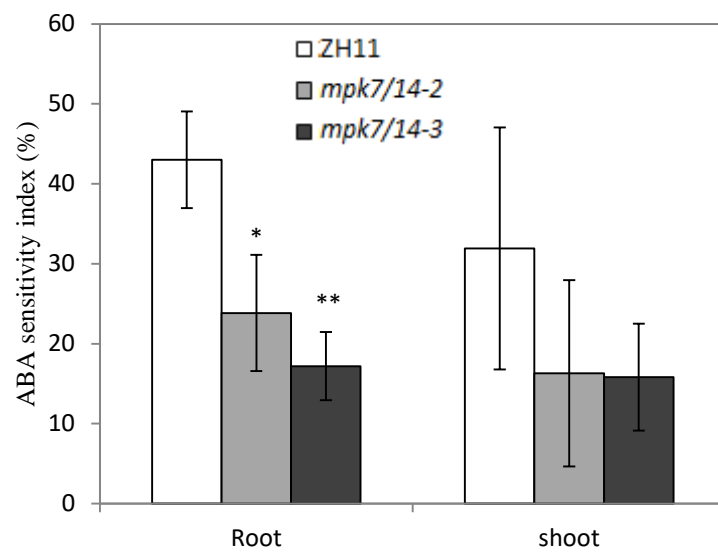

Supplement: Supplementary file 2 — Additional file 2: Fig. S2. Inhibitory effect of 0.2 μM ABA on mpk7/14. ABA sensitivity index (tissue length under 0.2 μM ABA treatment/length under 0 μM ABA treatment) of ZH11 and mpk7/14 for shoots and roots. Values represent the mean ± SD of three biological replicates (10 germinated seeds for each replicate). The asterisks indicate significant differences compared with ZH11. Student’s t-tests were used to generate p values (*p < 0.05, **p < 0.01). [file 12284_2023_679_MOESM2_ESM.pdf]
