# Supplementary material for: MKK3 Cascade Regulates Seed Dormancy Through a Negative Feedback Loop Modulating ABA Signal in Rice
Source: Rice (N Y). 2024 Jan 3;17:2. doi: 10.1186/s12284-023-00679-4 (PMC10764673; doi:10.1186/s12284-023-00679-4)
Supplement: Supplementary file 3 — Additional file 3: Table S1. Primers used in this study. [file 12284_2023_679_MOESM3_ESM.docx]

Table S1: The primers used in this paper

| Primer Name | Sequence(5'-->3') |
| --- | --- |
| **Transgenic vector construction** | |
| OsMKK3F | gggtaccggcgcgccaagcttATGGCGGGGCTCGAGGAGga |
| OsMKK3R | ataacgcgtactagtaagcttTCAGGCTTGGATGATGTATAGATCTT |
| OsMFT-6aF | CGGTATGTTAACCACGATCcggcagccaagccagca |
| OsMFT-6aR | GATCGTGGTTAACATACCGgttttagagctagaaat |
| OsMKK3-6bF | ACGTGGTAAGCACGGGCGCGcaacacaagcggcagc |
| OsMKK3-6bR | CGCGCCCGTGCTTACCACGTgttttagagctagaaat |
| **Detection of the knockout lines** | |
| OsMKK3-decF | AAGGCTCAGCAAACAAAGGTCA |
| OsMKK3-decR | AGCAGAGGAAGCAGCCAAGC |
| MFTdecF | CAGGTCTCCATCACTCCTCCAT |
| MFTdecR | AGGTAATGCATAGACAGGCAAC |
| **Subcellular localization** | |
| OsMKK3gfpF | caggaattcgatatcaagcttATGGCGGGGCTCGAGGAG |
| OsMKK3gfpR | gtcgacggtatcgataagcttGGCTTGGATGATGTATAGATCTTGC |
| OsMPK7gfpF | acggcatggacgagctgtacatggcgatgatggtggaccctc |
| OsMPK7gfpR | aatgtttgaacgatctgcagtcacatattcactcctgcaacaacctctg |
| **Luciferase complementation** | |
| MPK7NlucF | CGGGGGACGAGCTCGGTACCATGGCGATGATGGTGGACC |
| MPK7NlucFR | GCGTACGAGATCTGGTCGACCATATTCACTCCTGCAACAACCTC |
| PP2C50ClucF | ACGCGTCCCGGGGCGGTACCGAG GTG ATC TTG GTG ATGGCG |
| PP2C50ClucR | CGAAAGCTCTGCAGGTCGACGTTATGAAATGTTCTTGAGTTTCTTTCG |
| PYL7ClucF | ACGCGTCCCGGGGCGGTACCCATGAACGGCGCTGGTGGT |
| PYL7ClucR | CGAAAGCTCTGCAGGTCGACGATTGGCAAGGCGCTCCT |
| PYL11ClucF | ACGCGTCCCGGGGCGGTACC CCCGGAGATGAACAGTGGC |
| PYL11ClucR | CGAAAGCTCTGCAGGTCGACATTAACAAGGACTGGCAAGGCT |
| **Y2H** | |
| MPK7E1F | GACTGAATTCTTGATGGCGATGATGGTGGAC |
| MPK7B1R | CTGAGGATCCGCCTGTCACATATTCACTCCTGC |
| SAPK8EF | CACAGAATTCAAGCCGCAGGTAGGTAGAACCC |
| SAPK8XR | TCATCTCGAGACCCTGCCCCTTCAGTTCT |
| PP2C6EF | GTGAGAATTCATGGAGGACGTGGCGGT |
| PP2C6BR | GTCTggatccTCTGTCACTGTTGCTGTTCATCA |
| PP2C50EF | GACTGAATTC GAG GTG ATC TTG GTG ATGGCG |
| PP2C50XR | tgcagctcgagTTATGAAATGTTCTTGAGTTTCTTTCG |
| PP2C68EF | GACAGAATTCATGTCGATGGCGGAGGTGT |
| PP2C68BR | TGTCGGATCCTCTCTCTACAAGGCGTTGCCTC |
| PYL1EF | CAGTGAATTCCGAGCGCTTGATACAATGCCGTAC |
| PYL1BR | GTATGGATCCTCGCCAGGCAGGCATATGCA |
| PYL2EF | CAGAGAATTCAGACGACGACGAGGAGAAGCT |
| PYL2EF | CAGAGAATTCAGACGACGACGAGGAGAAGCT |
| PYL3EF | GATCTGAATTCTTGAGGATGGTGGAGGT |
| PYL3BR | CAGTGGATCCGCAACAATCACCGGTCGAG |
| PYL4EF | CTCAGAATTCACGAAGGAGACAAGGAAACAGC |
| PYL4BR | CTACGGATCCTCGCAACAACGAGGGAAAA |
| PYL5EF | GTCAGAATTCATGGTGGGGCTTGTGGGA |
| PYL5BR | GTCTGGATCCTACTGTTCAAGTGGCGAGGTG |
| PYL6EF | ACTGGAATTCATGATGCCGTACACCGCTC |
| PYL6BR | GACAGGATCCGTAGCTATGATCTAGGCGGCG |
| PYL7EF | CATCGAATTCATGAACGGCGCTGGTGGT |
| PYL7BR | GTAAGGATCCGATTGGCAAGGCGCTCCT |
| PYL9EF | GTCAGAATTC AAA TCA ATGGAGGCGCACGT |
| PYL9BR | CAGTGGATCCGACGATTTATTGACGAGGCCT |
| PYL10EF | GACAGAATTCATGGAGCAGCAGGAGGAAGTG |
| PYL10BR | GTCAGGATCCAAAGAAAAAGGCTATTCCGCC |
| PYL11EF | GATTGAATTCCCGGAGATGAACAGTGGC |
| PYL11BR | GTCAGGATCCATTAACAAGGACTGGCAAGGCT |
| **Expression analysis** | |
| OsMPK7reF | TGAAACTACTGCGACACTTGCG |
| OsMPK7reR | TCGATGGAGTATCCCTGCTGA |
| OsMKK3reF | TTGGAGTGAGTGCTGGTTTGG |
| OsMKK3reR | TCTTTTGGTGGTGTTGGTGATG |
| ABA45reF | AGAGAGGGGACAGCCCGATG |
| ABA45reR | AGGCTCAGCTTCCCCATCGC |
| Bzip23reF | TGGAATTGGAAGCTGAGGTAGC |
| Bzip23reR | GAGTCCTCCGAAGGCAAATTC |
| Lea3reF | CGGCAGCGTCCTCCAAC |
| Lea3reR | CGGTCATCCCCAGCGTG |
| Lip9reF | CTCTTCGACAACCTCCTTGGC |
| Lip9reR | TCCTCCTTCTTTGGCTCTTCC |
| OSBZ8reF | AGAAGCAGCGGGCACATG |
| OSBZ8reR | ATCAGTCGCCAGACCAGAGC |
| Rab16AreF | GCTCCAGCTCAAGCTCGTCT |
| Rab16AreR | CTCCCATTCCATCATCCTCAG |
| Rab16BreF | AGCTCCAGCTCGTCGTCTGA |
| Rab16BreR | TTGATCTTCTCCTTGATTCCCTTC |
| TRAB1reF | TCCATCTTCTTCGGGAACTACG |
| TRAB1reR | CCCGCTGAATCCATTTGTCC |
| EF1α-F | TTTCACTCTTGGTGTGAAGCAGAT |
| EF1α-R | GACTTCCTTCACGATTTCATCGTAA |
